# A Systematic Review of the Utility of Intraoperative Navigation During Total Shoulder Arthroplasty

**DOI:** 10.7759/cureus.33087

**Published:** 2022-12-29

**Authors:** Khemerin Eng, Alistair Eyre-Brook, David W Shields

**Affiliations:** 1 Trauma and Orthopaedics, Northern General Hospital, Sheffield, GBR; 2 Trauma and Orthopaedics, Glasgow Royal Infirmary, Glasgow, GBR

**Keywords:** operative planning, navigation, glenoid, reverse, anatomic, shoulder arthroplasty

## Abstract

Total shoulder arthroplasty (TSA) has been demonstrated to successfully recover function to shoulders impaired by arthrosis and rotator cuff insufficiency. Long-term survival depends on the correct positioning of glenoid components and secure bone fixation. Computed tomography (CT)-based intraoperative navigation has proven to be an effective technique for successful TSA procedures. This paper presents a review of CT-based intraoperative navigation considering its advantages and disadvantages. The crucial factors that contribute to the success of this technique are glenoid component positioning, operative duration, and screw selection, which are detailed in this review.

## Introduction and background

The clinical outcomes of anatomic total shoulder arthroplasty (aTSA) and reverse total shoulder arthroplasty (rTSA) rely on accurate correction of deranged glenohumeral morphology [1-4]. It has been demonstrated that the position of the glenoid component is paramount to success in these procedures, resulting in early cut-out and bone resorption, scapular notching, or complete fixation failure [5-8]. While computed tomography (CT) is becoming a routinely used informative tool in the planning of the glenoid component in shoulder arthroplasty, accurate replication of a planned glenoid position remains a challenge [9-12]. Component stability is particularly paramount in reverse arthroplasty, in which the lever arms place large shear stresses on the bone-implant interface. Various implant designs exist to mitigate this biomechanical challenge, giving a variety of base plate geometries and surfaces which require rigid fixation to the native bone, often using locked and/or unlocked screws [7,13]. Although bone purchase with the glenoid component can be increased by maximising the length and width of the screw, this is predicated on an accurate wire, drill, and screw trajectory, which is challenging given limited bone stock, challenging visualisation and variations in scapular morphology [14-17].

Conventionally, the surgeon relies on estimating the glenoid version and hidden bone stock, having no accurate reference of scapular orientation. This combined with limited glenoid exposure makes it difficult to place glenoid components accurately. Subsequent malalignment results in an unbalanced kinematic environment, eccentric loading, disordered vector forces, and an increase in the contact pressure and edge loading, hindering the ultimate success of the shoulder arthroplasty procedure [5,6,18-21].

Computed tomography scanning-based navigated systems

Conventional surgical methods involve preoperative radiograph templates and an intraoperative implant system relying on the surgeon’s internal compass and their experience of anatomy and deformity [22]. Many groups reported computer-assisted surgery for arthroplasty in recent years, but they were mainly focused on hip and knee arthroplasty with limited reports on shoulder arthroplasty [23-25].

The combination of preoperative planning, intraoperative navigation, and patient-specific instrumentation (PSI) has improved accuracy and has considerably decreased the deviation as per preoperative planning and outliers [23].

The following review looks exclusively into the impact of CT-based navigation systems on shoulder arthroplasty.

## Review

Methodology

The authors (KE & AE-B) conducted a structured literature search for primary research comparing conventional and navigation-based techniques pertaining to TSA from database inception until December 2021, following the Preferred Reporting Items for Systematic Reviews and Meta-Analyses (PRISMA) guidelines [26]. The search strategy was as follows: “glenoid” OR “baseplate” OR “screw,” “reverse total shoulder arthroplasty,” “total shoulder arthroplasty,” “intraoperative navigation” OR “Computer navigation” OR “intra-operative imaging” OR “computer-assisted Surgery” OR “computer assisted” OR “CT-guided Navigation” OR “CT-Based Navigation.”

**Figure 1:**
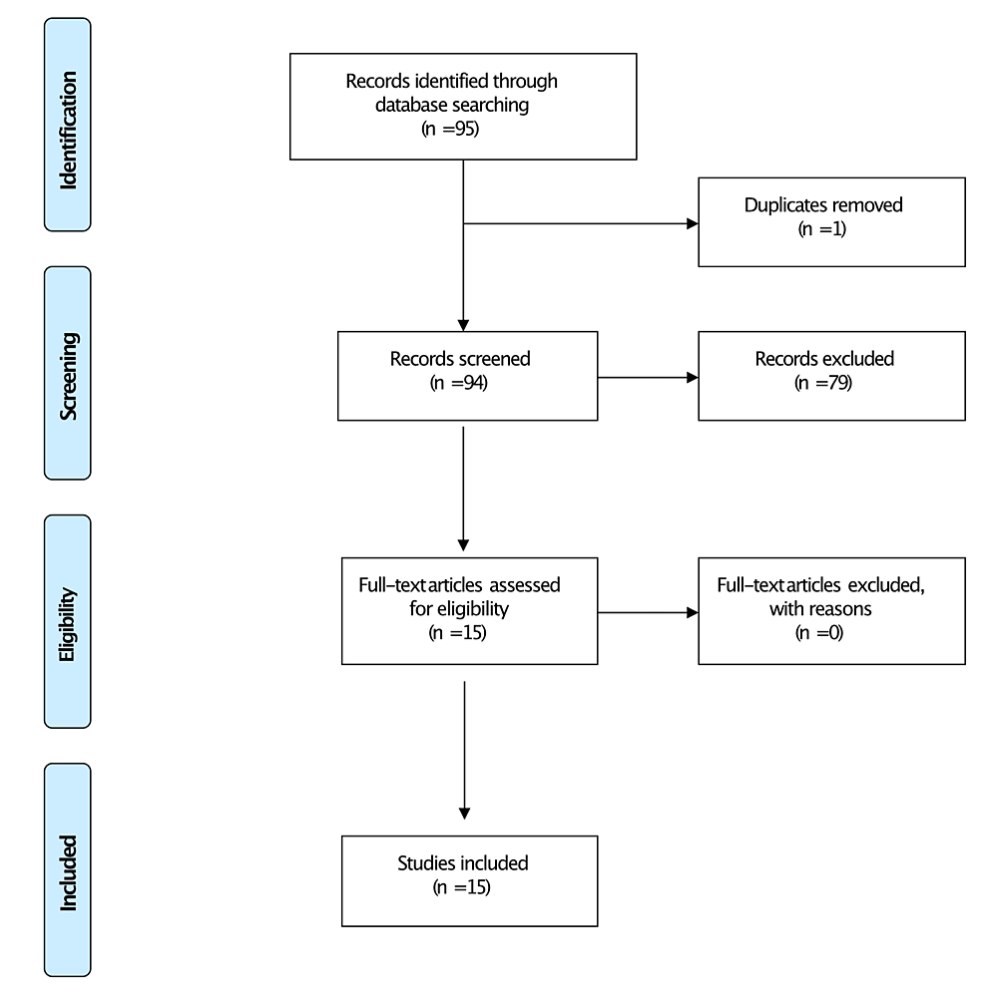
PRISMA flowchart. PRISMA = Preferred Reporting Items for Systematic Reviews and Meta-Analyses

Studies evaluating intraoperative navigation techniques using non-navigation as a comparator were included. We excluded studies not involving primary research data, without a comparator group, and without full text available. The authors performed a title and abstract review utilising the Rayyan interface [27]. Inclusion criteria were all articles explaining the advantages or disadvantages of CT-based navigation techniques in shoulder arthroplasty. Data were extracted using pre-piloted criteria from studies including glenoid component positioning, operating time, and impact on screw size and number. The search strategy returned 95 studies (Figure 1).

Following deduplication, title search, and abstract review, 15 studies met the criteria for inclusion in this narrative review (Table 1).

**Table 1:** Summary of studies reviewed. This table contains the relevant articles that explored both rTSA and TSA. The table also details the various outcomes measured. rTSA = reverse total shoulder arthroplasty; TSA = total shoulder arthroplasty; SPL = screw purchase length; 3D = three-dimensional

Author	Year	Study type	Patients	Implant	Measured outcome	Conclusions
Nashikkar et al. [5]	2019	Prospective case control	50	rTSA	SPL, screw angulation	Navigation contributes to increased stability via SPL
Nashikkar et al. [6]	2019	Prospective case control	60	TSA	Postoperative glenoid version and inclination	3D planning and navigation reduces the risk of glenoid placement deviation
Verbogt et al. [7]	2011	Cadaveric study	14	rTSA	Postoperative glenoid version and tilt	Navigation contributes to reduced tilt and version
Kircher et al. [22]	2009	Prospective randomised clinical trial	20	TSA	Operative time, glenoid version	Navigation increases glenoid positioning accuracy and greater retroversion correctional values
Nguyen et al. [18]	2009	Cadaveric randomised control trial	16	TSA	Initial guide pin placement, glenoid inclination, and version	Conventional instrumentation associated with higher errors in version and inclination
Wang et al. [23]	2020	Prospective case series	24	rTSA	Operative time, glenoid placement, complications	Navigation led to increased surgical time, approx. Eight cases to achieve competency in navigation compared to non-navigation for inclination placement
Rosenthal et al. [28]	2019	Retrospective cohort	200	Anatomic and reverse	Implant selection (augmented vs. non-augmented), operative time	Navigation increases the types of augmented glenoid components and increases operative time
Schoch et al. [29]	2020	Retrospective cohort	50	Anatomic and reverse	Glenoid version, inclination, fellow vs. trainee	High amount of mispositioning despite preoperative planning for both fellows and trainees
Hones et al. [30]	2021	Retrospective cohort	200	rTSA	Screw length and number of screws for baseplace	Navigation decreased required screw placement
Sprowls et al. [31]	2020	Retrospective case control	108	rTSA	Screw length, total number of screws and number of augmented baseplates	Navigation resulted in higher screw length, higher frequency of only two screw totals, higher augmented baseplates, as well as operative time
Gavaskar et al. [32]	2013	Case study	1	rTSA	Postoperative glenoid version	Navigation contributes to increased stability via SPL
Venne et al. [33]]	2015	Cadaveric study	30	rTSA	Screw endpoint and entry-point position. Baseplate position. Implantation time for operation	Navigation showed greater screw endpoint accuracy and slightly better precision
Moreschini et al. [34]	2020	Retrospective case study	40	rTSA	Screw length, two screw total, augmented baseplates	Navigation resulted in higher screw length, greater number of two screw totals, and greater use of augmented baseplates
Dekker and Tamble [35]	2020	Retrospective case series	2	rTSA	Glenoid version, bone graft size	Navigation allowed for accurate glenoid component version and screw placement. Longer operative time
Wanner et al. [8]	2019	Literature review	31	TSA and rTSA	Glenoid placement, preoperative planning	Introduction to navigation versus conventional

Nashikkar et al. sought to determine whether CT-based navigation improved the deviation of postoperative version and inclination compared to the manual technique. They found that computer-assisted glenoid components were within 50 of the preoperative plan in 81.8% of cases for version and 75.8% for inclination [6].

In a second paper, Nashikkar et al. also explored whether CT-based navigation improved glenoid baseplate fixation by either increasing screw purchase length and angulation or decreasing central cage perforation in patients undergoing rTSA [5,6]. They found that computer navigation contributed to significant alterations in SPL (anterior inadequate screw purchase length 64.7% vs. 95.2%), angulation, and central cage perforation (17.7% vs. 52.4%) compared to non-navigated methods. The average SPL increased and the screw angled more superiorly on average compared with the manual group.

In 2020, Verborgt et al. compared the accuracy of glenoid placement with computer navigation against those without for reversed shoulder arthroplasty [7]. The computer-assisted procedures discovered no complications relating to the positioning of coracoid process fixation pins. Glenoid version, tilt (range of error 8° vs. 16°), and screw siting were more accurate in the computer navigation group compared to the traditional instrumentation group.

Kircher et al. explored the utility of a CT-based navigation system for routine TSA and investigated the possibility of improved component orientation in the transverse plane [22]. The investigation showed overall improvement in accuracy in the positioning of the glenoid component in the transverse plane in the navigation group with greater values of correction to neutral retroversion (15.4° ± 5.8 to 3.7° ± 6.3 vs. 14.4° ± 6.1 to 10.9° ± 6.8).

The primary focus of the report by Nguyen et al. was to deliberate which steps of the glenoid resurfacing sequence were most susceptible to error [18]. They concluded that navigation methods are more efficient, but their CT measurements utilised non-standardised patient positioning during scans and were susceptible to rotation errors.

Wang et al. evaluated the learning curve of using computer navigation in glenoid implantation during rTSA. They compared the total surgical time in 24 consecutive patients operated on by a single surgeon [23]. Overall, they concluded that intraoperative computer navigation of glenoid component implantation did not increase the total surgical time for rTSA. Specifically, they found that the computer-assisted procedure requires less intraoperative time than the conventional approach (77.3 ± 11.8 minutes vs. 78.5 ± 18.1 minutes). Ultimately, they defined eight operative cases as the estimate to achieve competency in utilising intraoperative computer navigation for glenoid components.

In 2020, Rosenthal et al. sought to determine whether intraoperative three-dimensional (3D) planning affected glenoid component selection and operative time [28]. They retrospectively studied 100 consecutive patients using two-dimensional (2D) planning and compared it with another 100 consecutive patients in which 3D planning was used. Their findings demonstrated that intraoperative navigation required a slightly longer operating time; however, is typically used for a more diverse array of glenoid components with 3D planning (106.44 ±15.23 minutes vs. 117.9 ± 18.7 minutes).

Schoch et al. assessed the in vivo ability to implement preoperative planning using traditional instruments and commercial navigation software [29]. In this study, the surgeons were unable to reliably execute the placement of a glenoid component even after 3D preoperative planning using commercially available software. They advised considering other factors such as intraoperative navigation, intraoperative fluoroscopy and not 3D preoperative planning using standard instruments, and a central guide pin alone.

To explore the outcome of computer navigation on screw length and number during baseplate placement, Hones et al. evaluated their effect with rTSA placed in vivo [30]. They reported notably fewer screws per case (three screws vs. four screws on average, p < 0.001) and greater average screw length (35.0 mm in the navigation group vs. 32.6 mm in the conventional group, p < 0.001).

Sprowls et al. evaluated the impact of preoperative templating and intraoperative navigation on baseplate component specifications, including screw measurement length, screw number, and amount of augmented baseplate use [31]. Intraoperative computer navigation allowed for longer individual screw lengths (mean 36.7 mm vs. 30 mm), increased composite screw length, and fewer total screws used (frequency of using two screws total 68.6% vs 50.8% in non-navigated). They used Exactech for all cases.

In a case report of a rheumatoid patient with rotator cuff arthropathy, the use of a CT-guided rTSA was evaluated by Gavaskar et al. [32]. They found that CT navigation can help in improving screw length and purchase which may add to long-term stability. Real-time 3D feedback on the quality of glenoid bone provided optimal initial fixation of the glenoid component.

Venne et al. described a system for performing computer-assisted rTSA, including preoperative planning, intraoperative navigation, and postoperative evaluation, and then compared this with a conventional approach [33]. In their work, both accuracy and precision for screw angulation, the endpoint of screw placement (navigated 3.7± 2.4 mm vs. 9.7 ± 6.9 mm), and the endpoint of the baseplate central peg was improved in the navigated group compared to the conventional group which facilitated an improved bone purchase and a more secure glenoid implant fixation. They defined accuracy as how close the desired endpoint length was able to be replicated, and, therefore, the precision of repeated measurements under the same conditions.

When exploring screw numbers in a retrospective cohort, Moreschini et al. found statistically significant fewer amount of cases requiring greater than two screws to obtain a stable fixation in the navigation group compared to the conventional group (17 cases vs. nine cases, p = 0.019) [34]. The navigation group had, on average, a longer length of screw compared to the conventional group (35.5 ± 4.4 mm vs. 29.9 ± 3.6 mm; p < 0.001). They also found a large difference in augmentation utilisation between navigation versus conventional groups (13 vs. four cases, p < 0.009). Their study demonstrated the potential benefits of combining intraoperative navigation with CT pre-planning by allowing a fewer number of screws used to achieve primary stability. The factors such as screw number and overall alignment were found to be less significant in obtaining universal stability.

Dekker and Tambe conducted a retrospective case series study reviewing the utility of computer-based navigation on the accuracy of glenoid placement in rTSA [35]. They found that employing this form of intraoperative navigation allowed preferable baseplate implantation and screw hold, with successful postoperative function for the patient.

Wanner et al. reviewed the importance of anatomic restoration in shoulder arthroplasty [8] by exploring various forms of preoperative imaging, intraoperative guidance, and different modalities on the impact of accurate anatomic replacement. They found that CT navigation not only allowed for greater version/inclination assessment but also presented the chance for real-time adjustments intraoperatively. They found multiple studies demonstrating higher levels of accuracy with intraoperative navigation, including a study by Levy et al. that demonstrated starting point accuracy within 1.2 ± 0.7 mm of their preoperative goal, and inferior inclination and glenoid version within 1.2 ± 1.2 degrees and 2.6 ± 1.7 degrees, respectively [8]. Similarly, Walch et al. had a starting point accuracy within 1.05 ± 0.21 mm and inclination and version of 1.42 ± 1.37 degrees and 1.64 ± 1.01 degrees respectively [8].

Glenoid component positioning

The accurate positioning of glenoid components has been one of the key criteria for understanding intraoperative navigation advantages in shoulder arthroplasty. Misalignment leads to eccentric loading, the shift of vector forces, increased contact pressure and rim-loading, and increased stress at the implant-bone or bone-cement interface and the glenoid bone stock, and, therefore, potentially to an increased rate of glenoid failure. Computer-assisted navigation produces better accuracy in glenoid placement relative to non-navigation techniques. Seven studies were identified exploring the impact of intraoperative navigation on glenoid component positioning. Kircher et al. [22] found an overall improvement in accuracy with an average postoperative retroversion angle change from 15.40 ± 5.80 (range: 3.00-24.00) to 3.70 ± 6.30 (range: 8.00-15.00) in the navigation group and from 14.40 ± 6.10 (range: 2.00-24.00) to 10.90 ± 6.80 (range: -0.00-19.00) in the conventional group. Furthermore, the application of computer navigation in this setting may result in better prosthetic longevity and decreased need for revision procedures [7,22]. Recent studies have shown that navigation can improve the accuracy of placement of the inferior tilt of the glenoid component [19,36], which was not possible in the past [20].

Operating time

A potential disadvantage to the institution of routine intraoperative navigation is the added time and resource cost of using additional techniques. This is not a straightforward confounder as learning curves or the surgeon and theatre set-up time for additional devices must be considered, which will be subject to wide variation. Four studies evaluated the additional time associated with implementing navigated techniques. Wang et al. [23] undertook a prospective case series and concluded that eight operative cases will give a surgeon enough experience to develop competency with computer navigation for glenoid component factors with prior surgical training. Furthermore, they found that 23 CT-navigated cases were statistically similar to a matched group of 24 non-navigated, 77.3 minutes to 78.5 minutes, respectively, concluding that intraoperative computer navigation of glenoid component implantation does not increase the total surgical time for rTSA. The retrospective case series by Rosenthal et al. of 200 consecutive shoulder arthroplasty patients found that at the institution of the technique, intraoperative navigation slightly increased the duration of surgery, but this became insignificant as part of a learning curve within six months [28]. Based on the results of these studies, it is likely that, when compared with standard surgical techniques, intraoperative computer navigation will not demand a significantly extended amount of added operative time.

Impact on screw size and numbers

The number of screws can not only significantly alter glenoid bone reserve but also lead to a decreased operative time and a smaller risk of failure from glenoid stress risers [5,30,31,34,36]. Hones et al. reviewed 200 rTSAs, both navigated and conventional, and found that CT navigation utilised fewer screws and a greater length than the conventional technique: 3.4 vs. 4.1 screws, 35.0 mm vs. 32.6 mm screws, respectively [30]. Although limited in long-term clinical outcomes given the recent rise of navigation applications, these measurements can also impact the operative time and operative costs per case.

Operative time

This study outlines inconsistent reporting of computer-assisted navigation time of operative time. While Kircher et al. (169.5 ± 15.2 minutes vs. 138 ± 18.4 minutes), Sprowls et al. (98.6 ± 19.5 minutes vs. 85.8 ± 18.7 minutes), and Rosenthal et al. (11 minutes longer for intraoperative navigation) found that navigation time was significantly higher in the navigated group. Wang et al. (77.3 ± 11.8 minutes vs. 78.5 ± 18.1 minutes) and Venne et al. (23.5% less intraoperative time) found that the computer-assisted procedure requires less intraoperative time than the conventional approach.

Baseplate orientation error

A previous reported the comparative less baseplate orientation error in the case of computer-assisted navigation compared to the conventional group. Verborgt et al. found a range of error for version reduced for the navigation versus the non-navigated group (80, SD = 3.3 compared to 120, SD = 4.1). For tilt, the range of error was 80 (SD = 3.6) in navigated specimens and 160 (SD = 6.0) for controls. Nguyen et al. demonstrated that the mean absolute error in the glenoid version was reduced for the navigation method compared to the traditional technique (1.5 ± 1.90 vs. 7.4 ± 3.80, respectively, p < 0.05). Schoch et al. found the mean variation in version was 6.4 ± 5.6, and the mean variation in inclination was 6.6 ± 4.9.

Screw length

Maximizing the purchase length of the fixation screws dictates the initial stability of the glenoid baseplate in reverse shoulder arthroplasty. Although variations were observed in absolute reported screw length in this synthesis (both navigation and conventional cases), studies found longer screw lengths in the navigation groups compared to the conventional group. Nashikkar et al. (anterior 20 mm vs. 15 mm, p < .01) and posterior screws 20 mm vs. 13 mm, p < 0.01), Hones et al. (35.0 mm vs. 32.6 mm, p < 0.001), Sprowls et al. (36.7 mm vs. 30 mm, p < 0.0001) all reporting longer screw length for the navigation group.

Number of screws

A further advantage of using the navigation method is the use of fewer screws compared to the conventional method. Using fewer screws can preserve the glenoid bone supply, reduce stress risers, and shorten the operative duration. Schoch et al. detailed the total number of screws used in RSAs without computer navigation was 3.4 screws per case with navigation versus 4.1 screws with conventional placement (p < .001). Similarly in Sprowls et al., navigation resulted in the use of fewer screws (2.5 ± 0.7 vs. 2.8 ± 1, p = .047). For optimal fixation, the number of screws varied between two and five in different studies. This is attributable to the varying implant designs and surgeons’ estimation of adequate baseplate stability.

Complications

While the studies by Verborgt et al. and Kircher et al. found no intraoperative or postoperative complications even after the six-week follow-up, Wang et al. observed two complications, one with coracoid tracker malfunctioning, and in another case inadequate fixation of the coracoid tracker. Interestingly, Dekker et al. observed some disadvantages in the navigation method cautioning that the navigation system relies on accurately registered anatomic landmarks; therefore, malpositioned, malregistered, or subsequently loosened tracking devices will invariably produce incorrect data.

Disadvantages

The studies explored in this review demonstrate that CT-based navigation methods have a clear advantage in the treatment of shoulder arthroplasty compared to conventional methods. Still, this technique has limitations, and there is a scope for many improvements. For example, the need for an in-date preoperative CT and associated metal artefact in revision cases can complicate software protocols. Navigation techniques typically cease at guidewire positioning, and glenoid reaming is not involved in intraoperative imaging. The humeral component is paramount for stability, deltoid efficiency and joint loading, factors that can be neglected in intraoperative navigation studies. Although there has not been a significant difference in operating time between the navigation method and the conventional method, it can be further improved by reducing time in the case of the navigation method. Although 3D image intensifier algorithms have been used to reduce the effect, there is still room for refinement.

Limitations

The authors of these studies have unanimously concluded that navigation methods are superior compared to conventional methods but these studies have their own limitations. Nashikkar et al. and Moreschini et al. performed non-randomized case-control studies and did not define detailed comparisons of glenoid and scapula anatomy, which may affect screw placement. Similarly, in the study by Hones et al., factors such as acceptable screw fixation and the decision to implement more screws for fixation are subject to the surgeon’s judgement. The surgeon’s discretion and experience clearly bias the outcomes studied, particularly screw count and length.

## Conclusions

CT-based preoperative planning and intraoperative navigation may reduce the risk of glenoid placement outside of a neutral position and offers improved accuracy and more precise glenoid positioning in the transverse plane by decreasing the incidence of central perforation, increasing the average SPL and decreasing the incidence of inadequate screw purchase. Furthermore, the navigation-based technique for shoulder-based arthroplasty potentially impacts prosthetic longevity and revision rates; however, this remains to be proven in longitudinal studies. Intraoperative navigation slightly lengthens the duration of surgery which may be further improved with ongoing surgeon adoption and advancements in techniques. Multiple studies have demonstrated immediate benefits to using navigation; however, long-term clinical outcomes should remain the primary scope for future research.
